# Feasibility of 2-dimensional speckle tracking echocardiography strain analysis of the right ventricle with trans-thoracic echocardiography in intensive care: a literature review and meta-analysis

**DOI:** 10.1186/s44156-023-00021-0

**Published:** 2023-07-20

**Authors:** James McErlane, Ben Shelley, Philip McCall

**Affiliations:** 1 Anaesthesia, Critical Care & Peri-Operative Medicine Research Group, University of Glasgow, Room 2.73, 2nd Floor New Lister Building, 10-16 Alexandra Parade, G31 2ER Glasgow, UK; 2 Department of Anaesthesia, Golden Jubilee National Hospital, Clydebank, UK

**Keywords:** Right ventricle, Echocardiography, Speckle tracking, Feasibility, Intensive care

## Abstract

**Objectives:**

To identify variables that affect the feasibility of 2-dimensional right ventricular speckle tracking echocardiography (RV-STE) in the intensive care unit.

**Background:**

Trans-thoracic echocardiography (TTE) of the right ventricle is challenging. RV-STE is a novel echocardiography method thought to measure global RV function more fully than conventional TTE parameters. The feasibility of RV-STE in ICU populations has not been well described, and variables influencing RV-STE in ICU have not been investigated. This study aimed to address this.

**Methods:**

A literature review using Ovid MEDLINE(R) was undertaken. We performed meta-analysis with subgroup analysis of; RV-STE type (RV free-wall [RVFWLS] versus RV global longitudinal strain [RVGLS]), study design (prospective versus retrospective), coronavirus disease-19 (COVID-19) study or not, and strain software used. This was followed by meta-regression of proportion of invasive mechanical ventilation (IMV), with and without COVID-19 studies as a co-variate.

**Results:**

Eleven relevant studies from the literature search were identified, reporting an overall feasibility of RV-STE of 83.3% (95%CI 74.6–89.4%). Prospective study design was associated with higher feasibility compared with retrospective studies (p = 0.02). There were no statistical differences on univariate analysis between RVFWLS versus RVGLS, COVID-19 study or not, or strain software used. Meta-regression with COVID-19 study as a covariate demonstrated that higher proportions of IMV were significantly associated with worse feasibility (p = 0.04), as were COVID-19 studies (p < 0.01).

**Conclusions:**

We have identified three variables associated with poor feasibility; retrospective study design, COVID-19 studies, and proportion of IMV. A prospective study design should be viewed as gold standard to maximise RV-STE feasibility.

**Supplementary Information:**

The online version contains supplementary material available at 10.1186/s44156-023-00021-0.

## Feasibility of 2-dimensional speckle tracking echocardiography strain analysis of the right ventricle in intensive care: a literature review and meta-analysis

Right ventricular (RV) dysfunction is prevalent, and associated with poor outcomes in critically ill patients in the intensive care unit (ICU) [1]. Owing to its portability, low-cost, and widespread availability, the mainstay of RV functional assessment is by trans-thoracic echocardiography (TTE). However, its complex geometry, irregular shape, retrosternal position, and marked load dependence, makes RV TTE assessment notoriously challenging. Quantitative analysis of RV function often relies on extrapolation of data from a single ventricular region (such as tricuspid annular plane systolic excursion and S’ wave velocity at the tricuspid annulus) as being representative of the function of the whole ventricle. Two-dimensional speckle tracking echocardiography (STE) longitudinal strain analysis is thought to overcome some of these limitations, and has been shown to have better correlation with gold standard cardiac magnetic resonance RV ejection fraction than TAPSE, S’, and RV fractional area change [2, 3]. Given the perceived superiority of RV-STE over conventional RV echocardiography parameters, it was selected as the focus of this review. RV-STE strain analysis is however reliant on acquiring a high-quality image of the RV free wall which can be difficult to obtain in the ICU patient population.

To identify factors that might affect feasibility of RV-STE strain analysis in ICU, and determine strategies that might improve feasibility, we performed a literature review and meta-analysis. Feasibility was defined as the percentage of echocardiography studies with images of sufficient quality to allow RV-STE strain analysis. A literature search was performed using Ovid MEDLINE(R) (Additional file 1: Figs. S1, S2), identifying 11 ICU studies that reported RV-STE feasibility.

**Fig. 1:**
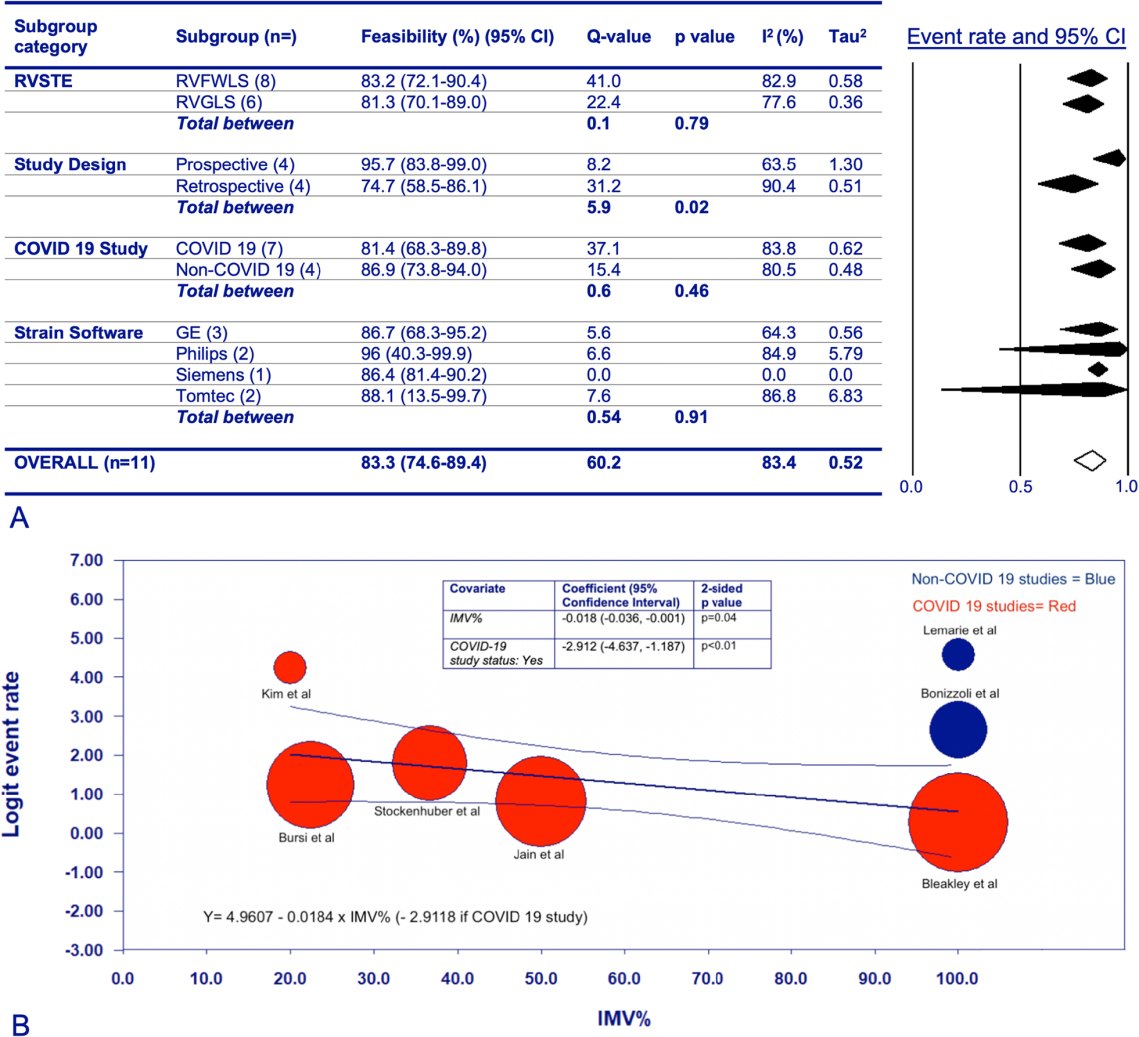
Subgroup meta-analyses, and meta-regression of the feasibility of RV-STE in ICU. **A** Subgroup meta-analyses of RV-STE feasibility in ICU with forest plot. Diamonds within the forest plot represent the point estimate and 95% confidence interval. A random effects model was used for between groups comparison. **B** Meta-regression of RV-STE feasibility. Proportion of patients receiving IMV included as a continuous variable, with COVID-19 study status as a co-variate. RV-STE feasibility is described in terms of logit event rate. The bubble plot demonstrates the weight of each study in the meta-regression, with larger bubbles having greater weight

Meta-analysis was performed on these 11 studies using Comprehensive Meta-Analysis Version 4 (Biostat, Inc.). A random effects model was employed. In the overall meta-analysis, and subgroup meta-analyses, if both RV free-wall (RVFWLS) and global longitudinal strain (RVGLS) were reported, RVFWLS was used due to its more rigorous validation [2]. If RVFWLS wasn’t reported, RVGLS was used. The exception to this was the RVFWLS versus RVGLS subgroup meta-analysis, where if RVFWLS and RVGLS were both reported by a study, both were included. In the overall meta-analysis, RV-STE feasibility was 83.3% (95%CI 74.6–89.4%, Additional file 1: Fig. S3). Subgroup meta-analyses and meta-regression were performed to investigate variables affecting feasibility, including: RVFWLS versus RVGLS, study design (retrospective/prospective), ICU diagnosis (coronavirus disease-2019 [COVID-19] or not), strain software used, and proportion of patients receiving invasive mechanical ventilation (IMV) (Fig. 1A).

Subgroup meta-analysis did not show any statistical difference in feasibility between RVFWLS versus RVGLS. Prospective studies showed significantly higher RV-STE feasibility, and appeared to have lower heterogeneity, compared with retrospective studies (p = 0.02, I^2^ 63.5 vs 90.4 Fig. 1B). On univariate analysis there was no statistical difference between COVID-19 and non-COVID-19 studies, nor between strain software (recognising the substantial limitation of comparing four different software across eight studies).

The impact of IMV was analysed using meta-regression, with the proportion of patients receiving IMV as a continuous variable (Fig. 1B). We firstly conducted the meta-regression in patients with COVID-19 receiving IMV, showing that higher proportions of IMV are associated with poorer RV-STE feasibility (p = 0.03, Additional file 1: Fig. S4). Given our a- priori hypothesis that COVID-19 may impact feasibility, particularly in ventilated patients, we repeated the meta-regression with COVID-19 study status as a co-variate. Multivariate meta-regression determined that proportion of IMV (p = 0.04) and COVID-19 study status (p < 0.01) are independently associated with RV-STE feasibility. I^2^ demonstrates that 46.1% of the variability in the observed effects is attributable to true effects variability. R^2^ is 73%, showing that the majority of this variance in true effects is explained by the meta-regression model incorporating IMV proportion and COVID-19 study status as co-variates. We were unable to include additional co-variates in our meta-regression due to the small number of studies.

Meta-analysis and meta-regression have identified three factors that affect the feasibility of RV-STE strain analysis; study design, COVID-19 study status, and proportion of IMV. Prospective studies allow optimisation of imaging for STE strain analysis, leading to better feasibility. Lemarie et al. reported 100% feasibility for RV-STE in patients with ARDS undergoing IMV [4]. They used a prospective design, emphasising optimal patient positioning, and obtaining an RV focussed apical four-chamber view. Proportion of IMV and COVID-19 are both independent predictors of poor feasibility. In addition to the difficulty in optimally positioning sedated patients receiving IMV, high airway pressure and alveolar distension may impair acoustic windows leading to poor feasibility. Bursi et al. highlighted the struggle with “cumbersome PPE with limited scan time” when performing echocardiography in patients with COVID-19 [5], whilst in the conduct of our own multicentre study of RV function in patients with COVID-19 receiving IMV [6], attitudes to echocardiography varied dramatically between sites. “Echocardiography technicians not being allowed in ‘red zones’”, and “the allocation of poor quality echocardiography equipment to COVID-19 areas” being challenges reported.

Limitations of our study include the small number of studies available for meta-analyses and the variation of strain software used across the studies. Different strain software use different proprietary algorithms to derive strain and therefore will report slightly different strain results. Strengths of our study include that it addresses an important clinical subject with an overall aim to improve the feasibility of RV-STE analysis on ICU, additionally we used a comprehensive array of subgroup meta-analyses with meta-regression to elucidate factors affecting RV-STE feasibility.

As TTE is increasingly utilised on ICU, interest in RV-STE strain continues to intensify. A prospective study design should be viewed as gold standard to maximise feasibility. Prospective design affords optimal patient positioning, the ability to pause ventilation, and to mandate an RV focused view; modifiable factors that will enhance RV-STE feasibility.

## Supplementary Information

**Additional file 1: Figure S1.**Literature search strategy. **Figure S2. **Flow diagram of literature search. **Figure S3. **Overall Meta-analysis. **Figure S4. **Meta-regression: studies with COVID-19.

## Data Availability

The data used for this manuscript will be available from the corresponding author upon reasonable request.

## Abbreviations

COVID-19: Coronavirus disease-2019
ICU: Intensive care unit
IMV: Invasive mechanical ventilation
RV: Right ventricular
RVFWLS: Right ventricular free wall longitudinal strain
RVGLS: Right ventricular global longitudinal strain
STE: Speckle tracking echocardiography
TTE: Trans-thoracic echocardiography
