# Supplementary material for: Feasibility of 2-dimensional speckle tracking echocardiography strain analysis of the right ventricle with trans-thoracic echocardiography in intensive care: a literature review and meta-analysis
Source: Echo Res Pract. 2023 Jul 20;10:11. doi: 10.1186/s44156-023-00021-0 (PMC10357770; doi:10.1186/s44156-023-00021-0)
Supplement: Supplementary file 1 — Additional file 1: Figure S1.Literature search strategy. Figure S2. Flow diagram of literature search. Figure S3. Overall Meta-analysis. Figure S4. Meta-regression: studies with COVID-19. [file 44156_2023_21_MOESM1_ESM.docx]

Additional Material: Feasibility of 2-dimensional speckle tracking echocardiography strain analysis of the right ventricle with trans-thoracic echocardiography in intensive care: a literature review and meta-analysis

Contents

Figure S1. Literature Search Strategy Page 2

Figure S2. Flow Diagram of Literature Search Page 3

Figure S3. Overall Meta-analysis Page 4

Figure S4. Meta-regression: Studies with COVID-19

patients requiring IMV Page 4

Ovid MEDLINE(R) and In-Process, In-Data-Review & Other Non-Indexed Citations <1946 to October 07, 2021>

No. of search results

1 Critical Care/ 56105

2 critical care.mp. 78291

3 intensive care.mp. 193284

4 icu.mp. 65421

5 intensive therapy.mp. 5083

6 itu.mp. 986

7 sepsis.mp. 133514

8 septic shock.mp. 24205

9 acute respiratory distress syndrome.mp. 17728

10 ards.mp. 15352

11 covid 19.mp. 151959

12 covid.mp. 152645

13 pulmonary embolism.mp. 55792

14 mechanical ventilation.mp. 47677

15 Positive-Pressure Respiration/ 17723

16 Ventricular Function, Right/ 7119

17 Ventricular Dysfunction, Right/ 6443

18 (right adj3 ventric*).mp. 70333

19 (echo* adj5 speckle).mp. 3945

20 (track* adj5 speckle).mp. 5785

21 (strain adj7 echo*).mp. 3452

22 deformation.mp. 50238

23 1 or 2 or 3 or 4 or 5 or 6 or 7 or 8 or 9 or 10 or 11 or 12 or 13 or 14 or 15 613683

24 16 or 17 or 18 70333

25 19 or 20 or 21 or 22 56231

26 23 and 24 and 25 168

Input and approval for the literature search strategy was received by Mr. Paul Cannon (University of Glasgow College Librarian) on 07/10/2021.

Figure S1. Literature Search Strategy

**Identification**

Studies identified with Ovid MEDLINE(R)

(n=168)

Abstracts reviewed

(n=168)

Studies excluded based on abstract

(n=143)

Studies excluded (n=14):

Number of ECHO scans excluded due to poor quality not described (n=7)

Combined LV and RV strain feasibility reported (n=4)

Studies using overlapping patient cohort (n=3)

Studies assessed for eligibility

(n=25)

**Screening**

Studies included in review

(n=11)

1. Baycan O, Barman H, Atici A, Tatlisu A, Bolen F, Ergen P, et al. Evaluation of biventricular function in patients with COVID-19 using speckle tracking echocardiography. *The International Journal of Cardiovascular Imaging*. 2021;37(1).

2. Bleakley C, Singh S, Garfield B, Morosin M, Surkova E, Mandalia M, et al. Right ventricular dysfunction in critically ill COVID-19 ARDS. *International Journal of Cardiology*. 2021;327.

3. Bonizzoli M, Cipani S, Lazzeri C, Chiostri M, Ballo P, Sarti A, et al. Speckle tracking echocardiography and right ventricle dysfunction in acute respiratory distress syndrome a pilot study. *Echocardiography*. 2018;35(12).

4. Bursi F, Santangelo G, Sansalone D, Valli F, Vella A, Toriello F, et al. Prognostic utility of quantitative offline 2D-echocardiography in hospitalized patients with COVID-19 disease. *Echocardiograph*y. 2020;37(12).

5. Dahhan T, Siddiqui I, Tapson V, Velazquez E, Sun S, Davenport C, et al. Clinical and echocardiographic predictors of mortality in acute pulmonary embolism. *Cardiovascular Ultrasound*. 2016;14(1).

6. Jain R, Salinas PD, Kroboth S, Kaminski A, Roemer S, Perez Moreno AC, et al. Comprehensive Echocardiographic Findings in Critically Ill COVID-19 Patients With or Without Prior Cardiac Disease. *Journal of Patient-Centered Research and Review*s. 2021;8(1).

7. Khemasuwan D, Yingchoncharoen T, Tunsupon P, Kusunose K, Moghekar A, Klein A, et al. Right ventricular echocardiographic parameters are associated with mortality after acute pulmonary embolism. Journal of the American Society of Echocardiography: Official Publication of the American Society of Echocardiography. 2015;28(3).

8. Kim M, Nam J, Son J, Kim S, Son N, Ahn C, et al. Cardiac Manifestations of Coronavirus Disease 2019 (COVID-19): a Multicenter Cohort Study. *Journal of Korean Medical Scienc*e. 2020;35(40).

9. Lemarié J, Maigrat CH, Kimmoun A, Dumont N, Bollaert PE, Selton-Suty C, et al. Feasibility, reproducibility and diagnostic usefulness of right ventricular strain by 2-dimensional speckle-tracking echocardiography in ARDS patients: the ARD strain study. *Ann Intensive Care*. 2020;10(1).

10. Li Y, Li H, Zhu S, Xie Y, Wang B, He L, et al. Prognostic Value of Right Ventricular Longitudinal Strain in Patients With COVID-19. *J Am Coll Cardiol Im*. 2020;13(11).

11. Stockenhuber A, Vrettos A, Androschuck V, George M, Robertson C, Bowers N, et al. A pilot study on right ventricular longitudinal strain as a predictor of outcome in COVID-19 patients with evidence of cardiac involvement. Echocardiography. 2021;38(2).

Figure S2. Flow Diagram of Literature Search

**Included**


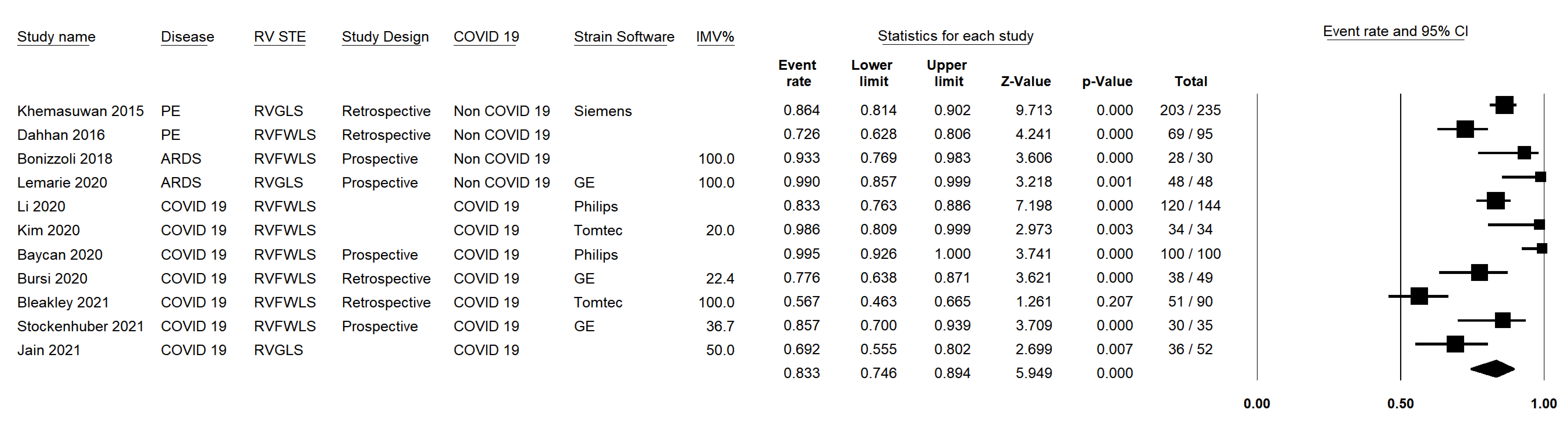


Figure S3. Overall Meta-analysis: Feasibility of 2-Dimensional Speckle Tracking Echocardiography of the Right Ventricle in Intensive Care.


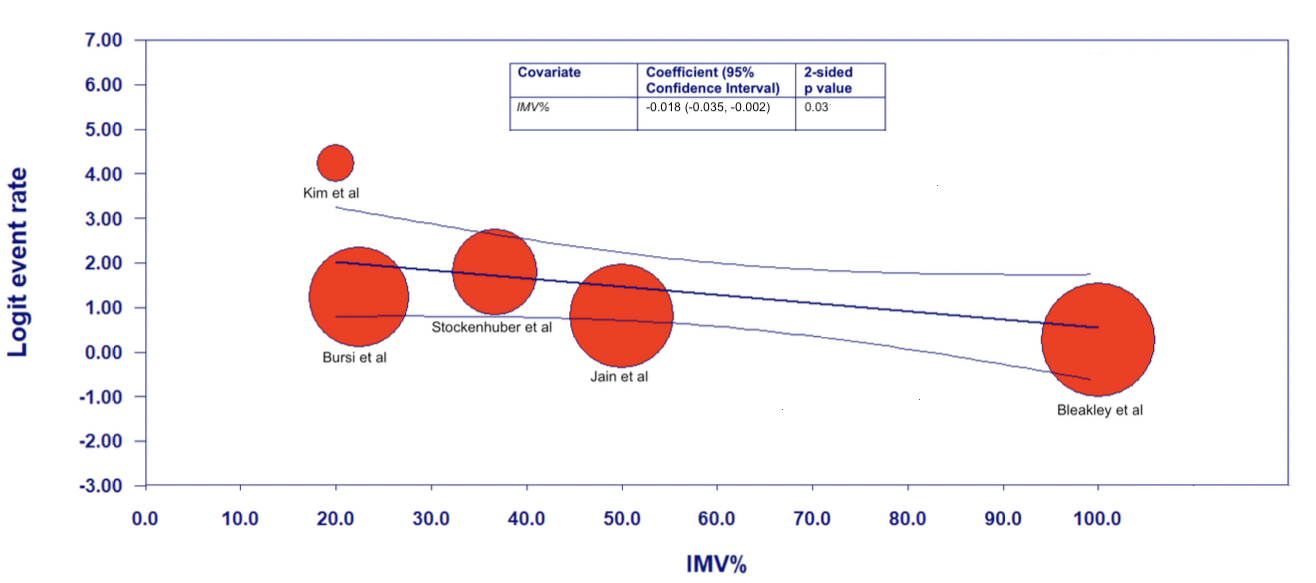


Figure S4. Meta-regression: Studies with COVID-19 patients requiring IMV
